# How Does Medical Device Regulation Perform in the United States and the European Union? A Systematic Review

**DOI:** 10.1371/journal.pmed.1001276

**Published:** 2012-07-31

**Authors:** Daniel B. Kramer, Shuai Xu, Aaron S. Kesselheim

**Affiliations:** 1 Harvard Medical School, Boston, Massachusetts, United States of America; 2 Cardiovascular Division, Beth Israel Deaconess Medical Center, Boston, Massachusetts, United States of America; 3 Division of Pharmacoepidemiology and Pharmacoeconomics, Department of Medicine, Brigham and Women's Hospital, Boston, Massachusetts, United States of America; Ottawa Hospital Research Institute, Canada

## Abstract

**Background:**

Policymakers and regulators in the United States (US) and the European Union (EU) are weighing reforms to their medical device approval and post-market surveillance systems. Data may be available that identify strengths and weakness of the approaches to medical device regulation in these settings.

**Methods and Findings:**

We performed a systematic review to find empirical studies evaluating medical device regulation in the US or EU. We searched Medline using two nested categories that included medical devices and glossary terms attributable to the US Food and Drug Administration and the EU, following PRISMA guidelines for systematic reviews. We supplemented this search with a review of the US Government Accountability Office online database for reports on US Food and Drug Administration device regulation, consultations with local experts in the field, manual reference mining of selected articles, and Google searches using the same key terms used in the Medline search. We found studies of premarket evaluation and timing (*n* = 9), studies of device recalls (*n* = 8), and surveys of device manufacturers (*n* = 3). These studies provide evidence of quality problems in pre-market submissions in the US, provide conflicting views of device safety based largely on recall data, and relay perceptions of some industry leaders from self-surveys.

**Conclusions:**

Few studies have quantitatively assessed medical device regulation in either the US or EU. Existing studies of US and EU device approval and post-market evaluation performance suggest that policy reforms are necessary for both systems, including improving classification of devices in the US and promoting transparency and post-market oversight in the EU. Assessment of regulatory performance in both settings is limited by lack of data on post-approval safety outcomes. Changes to these device approval and post-marketing systems must be accompanied by ongoing research to ensure that there is better assessment of what works in either setting.

*Please see later in the article for the Editors' Summary.*

## Abstract 2

Aaron Kesselheim and colleagues conduct a systematic review to examine the strengths and weaknesses associated with approaches to medical device regulation in the US and EU.

## Editors' Summary

**Background:**

Medical devices—health technologies that are not medicines, vaccines, or clinical procedures—cover a vast range of equipment from the simple to the more complex. Medical devices are essential for patient care, and in the past decade, new devices have offered improved treatment alternatives for many diseases and conditions, leading to substantial growth in the US$350 billion medical device industry. However, new medical devices also pose substantial risks to patients, as shown in recent high-profile product recalls involving breast implants and artificial hip implants.

**Why Was This Study Done?:**

Concerns about the safety of new medical devices have led to calls for greater testing of the safety and effectiveness of new devices before they come on the market and for improved monitoring of their performance after new devices have been approved for use by a regulatory body. In this study, the researchers systematically reviewed evidence about the performance of medical device approval and post-market surveillance systems in two of the most important world markets for medical devices—the United States and the European Union.

**What Did the Researchers Do and Find?:**

The researchers performed a keyword search in Medline (a database of published biomedical literature) for all relevant articles, and supplemented this search with a review of reports on Food and Drug Administration (FDA) device regulation in the US Government Accountability Office's online database. Then they consulted with both US and EU experts and also conducted Google searches to capture reports by management consultant firms. The researchers included only those studies that reported empirical data, either qualitative or quantitative, about the characteristics, performance metrics, or effectiveness of device evaluation or post-market oversight in the US or EU.

Using these methods the researchers identified nine studies that focused on pre-market evaluation and timing, eight studies of device recalls, and three surveys of device manufacturers. Because of the variable quality and lack of outcomes from these studies and reports, the researchers concluded that these studies offered only limited insights into either the US or EU systems. But the available evidence does suggest that in the US, the FDA could improve oversight of device approval, for example, by following up on its commitment to reclassify high-risk medical devices and improve post-market surveillance of devices that are approved on the basis of limited data. The researchers also suggest that using recalls to measure the safety record of individual devices or classes of devices is flawed, as particular devices may be over- or underrepresented in recall data depending on the frequency of their use, design complexity, and the clinical manifestations of malfunction. In the EU, apart from a few studies addressing the timing of approval, the researchers found almost no robust data on device regulation. Some case reports suggested substantial dangers to patients in the EU from devices approved on the basis of limited data, but the researchers could not systematically compare the quality of studies used for device approval or post-approval safety outcomes between the EU and US, mainly because of the lack of transparency among the EU regulators (Notified Bodies).

**What Do These Findings Mean?:**

These findings show that few studies have quantitatively assessed medical device regulation in either the US or EU, but the existing studies examined in this review suggest that policy reforms are necessary for both device approval and post-market evaluation of performance, including improving classification of devices in the US and promoting transparency and postmarket oversight in the EU. However, assessment of regulatory performance in both the US and EU is limited by lack of data on post-approval safety outcomes. Any changes to medical device approval and post-marketing systems should be accompanied by ongoing research and evaluation to ensure that there is an improved assessment of what works in either setting.

**Additional Information:**

Please access these websites via the online version of this summary at http://dx.doi.org/10.1371/journal.pmed.1001276.

- This study is further discussed in a PLoS Medicine Perspective by Sanket Dhruva and Rita Redberg
- The WHO website has a comprehensive topic section on medical devices
- Information on medical devices is also available from the FDA and the European Commission

## Introduction

As medical devices play a growing role in the diagnosis and management of disease, the global medical device industry has surpassed US$350 billion in annual revenue [1]. In the past decade, new devices have offered improved treatment alternatives for cardiovascular, orthopedic, oncologic, and many other diseases. But new devices have also posed substantial risks to patients, with high-profile recalls in recent years affecting breast implants [2], specific types of artificial hips [3], devices for lung surgery [4], and implantable cardioverter-defibrillator leads [5]. These episodes have led experts to call for greater pre-market testing for safety and effectiveness of new devices and monitoring of their performance after approval [6],[7].

The United States (US) and the European Union (EU), two of the most important world markets for medical devices, present vastly different approaches to approving devices for use in patients [8]. In the US, approximately two-thirds of all newly marketed devices are exempt from formal evaluation by the US Food and Drug Administration (FDA), including most low-risk (Class I) devices such as stethoscopes. Most moderate-risk devices (Class II, such as computed tomography scanners) and some high-risk devices (Class III, such as pacemakers or replacement heart valves) are cleared by the FDA through the “510(k)” pathway, based on substantial equivalence to previously approved devices, without requiring clinical trials. The highest risk devices are supposed to be subjected to trials by the manufacturer testing clinical end points and approved by the FDA through a process called pre-market approval (PMA) [9]. PMA may include additional requirements such as bench and animal testing as well as clinical data designed to address safety and effectiveness. In the EU, medical devices subjected to the PMA process in the US may be approved—that is, granted a Conformité Européenne (CE) mark—by local organizations called Notified Bodies based on more limited pre-market testing merely showing that the devices work as intended in a manner where the benefits outweigh the risks. Devices subject to the EU process may be available to patients sooner, albeit with less clinical experience prior to use.

Dissatisfaction with both the US and EU device approval and post-market evaluation systems has reached a critical level [7],[10]–[12]. While the FDA has been criticized for its cumbersome requirements and delays in approval [11],[13], the system in the EU has been charged with failing to gather meaningful data [12]. Reforms are being considered in both environments, as US legislators debate the re-authorization of the Medical Device User Fee and Modernization Act (MDUFMA) [14] and the European Commission reviews the directives that regulate medical devices [15]. Empirical data regarding medical device regulation should inform policymakers in both settings. Thus, we sought to systematically identify studies of the performance of the US and EU medical device approval and post-market surveillance systems. Our goal was to understand the evidence basis supporting changes to each system and to identify, if possible, how current proposals regarding alterations to medical device oversight might affect patients, providers, industry, and public health in the US and EU.

## Methods

### Data Sources and Searches

**Figure 1:**
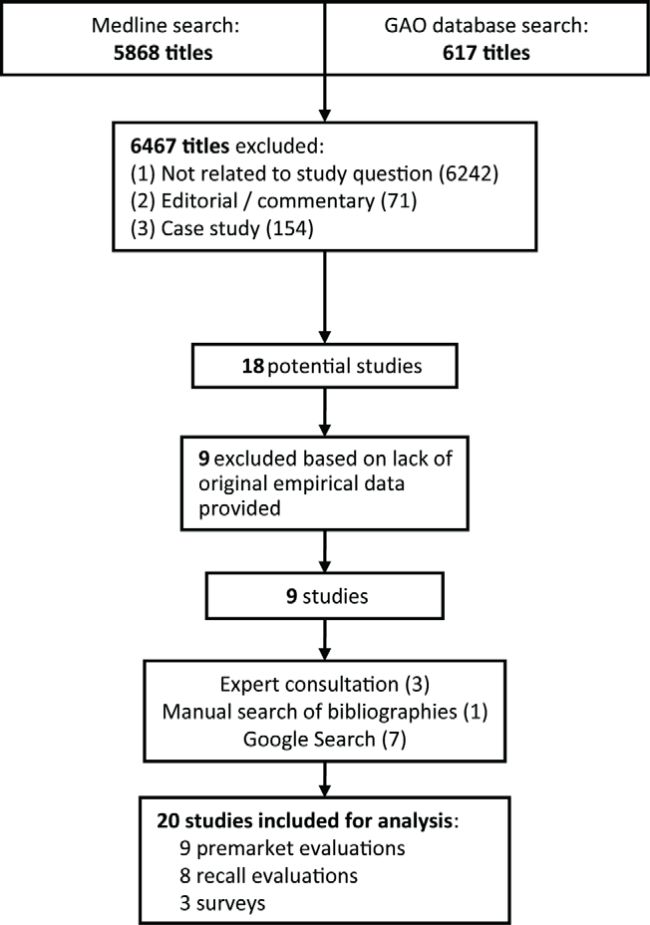
PRISMA schematic of systematic review search process.

Following PRISMA guidelines for systematic reviews (Text S1), to obtain our starting sample, we performed a Medline search for all articles listed prior to July 31, 2011 using two nested categories that included medical devices and glossary terms attributable to the FDA (e.g., “510(k)” and “PMA”) and the EU (e.g., “notified body” and “CE mark”). We supplemented this search with a review of the US Government Accountability Office (GAO) online database for reports on FDA device regulation using the same search terms. No language requirement was placed on the searches. After we obtained our initial sample, we used the titles and abstracts to identify potential studies; we then obtained the full articles to confirm which studies would be included in the systematic review (Figure 1).

When the Medline search was complete, we consulted with both US and EU experts in the field, engaged in manual reference mining of our sample of articles, and, finally, conducted Google searches using the same key terms used in the Medline search. Google searches were deemed necessary based on our discussions with local experts, from whom we learned that certain widely cited reports in this field were conducted by management consultant firms and directly disseminated as white papers, which would not be captured by Medline or other academic databases.

### Study Selection

Our prespecified inclusion criteria involved characteristics of the studies themselves, and the data presented. Studies published in peer-reviewed journals, reports disseminated as “white papers” in digital or other publicly accessible media, or reports presented in a public forum were eligible for inclusion. Case studies, editorials, opinion pieces, commentaries, and reviews or meta-analyses without original data or analysis were excluded.

Studies included must have reported empirical data about the characteristics, performance metrics, or effectiveness of device evaluation or post-market oversight in the US or EU. “Device evaluation” was defined as regulatory assessment of medical devices that occurred prior to marketing approval, while “post-market oversight” was defined as regulatory management of medical devices that occurred after approval. We did not assess studies addressing other parts of the world. The data could be gathered in a quantitative (e.g., rates of review times, rates of recalls, or objective characteristics of the evaluation process) or qualitative fashion (e.g., reports of surveys or focus groups). If not published in a peer-reviewed scientific journal, reports were required to have identifiable methodologies/analytic approaches, results, and discussion sections. Thus, routine statistical reports of raw data, such as numbers of submissions and approvals posted as part of FDA annual reporting, were excluded.

### Data Extraction and Analysis

Two authors (D. B. K. and S. X.) separately extracted data from the results sections of the included studies and categorized data based on the aspect of device regulation that was assessed. We noted the methodology and results, with particular focus on sample size, research choices that might influence outcomes (such as selection of denominators and calculations of medians versus means), and whether public health end points were assessed. We then reviewed the results and resolved differences by consensus among the three investigators. We formally assessed the major limitations of each study. Possible sources of bias in the included studies were noted, including funding sources, methodological limitations (including lack of detailed reporting of methods or study instruments such as surveys or interview guides), and use of peer review. Specific attention was paid to the data sources analyzed by in the studies, such as survey samples and publicly reported adverse event repositories. Because of the descriptive nature of the extraction, kappa values for correlation were not calculated. Given the heterogeneity of studies, we did not conduct a meta-analysis after qualitative data synthesis.

## Results

The initial Medline and GAO searches returned 6,485 hits, from which we ultimately identified nine to be included in our analysis (Figure 1). Manual reference mining and Google searches added an additional eight, and expert consultation added an additional three studies. See Table 1 for a full summary of each study or report identified.

**Table 1:** Studies assessing and/or comparing US and EU systems for approval and post-market surveillance of medical devices.

Article (Year)	System Studied	Methodology	Results	General Conclusions
**Studies of pre-market evaluation: empirical basis for device approval**				
Dhruva et al. [16] (2009)	US	Descriptive analysis of pre-market studies of cardiovascular devices performed for PMA process, 2000–2007	33 of 123 studies (27%) were randomized and 14% were blinded. 111 of 213 primary end points (52%) were compared with controls (31% retrospective). Of the primary end points, 88% were surrogate markers of effectiveness rather than “final” outcomes (e.g, mortality).	(1) Studies supporting cardiovascular PMAs lack some of the hallmarks of well-designed clinical trials, they may not accurately represent women, and the results may not be reported fairly.(2) All four studies focused on original PMA applications in one organ system, challenging generalizability.(3) Certain classic features of clinical trials, such as randomization and blinding, are not always possible in device trials.(4) Comparative studies not performed in EU due to lack of data availability.
Dhruva et al. [16] (2009)	US	[Same]	Gender not reported in 34 of 123 studies (28%); 41% of studies included analysis or comment on gender-specific outcomes.	
Chen et al. [17] (2011)	US	[Same]	Only 20 of 123 (16%) studies included “training patients” (the first individuals in whom a device is tested). All training patients were excluded from effectiveness analyses, and 95% were excluded from safety analyses, suggesting that the data submitted to FDA may be biased in favor of the device’s effectiveness and safety.	
Kramer et al. [20] (2009)	US	[Same]	Pivotal trial end points were of “high quality” for 82% of effectiveness end points and 60% of safety end points. Subject accounting was “high quality” in 77% of studies.	
**Studies of pre-market evaluation: timing of approval**				
Gollaher and Goodall [21] (2011)	US and EU	Descriptive analysis of length of pre-market evaluation and comparison of US versus EU time differences for convenience sample of devices	In the US, after 2007, mean approval times increased for devices approved via both PMA (by 75%, to 27 mo) and 510(k) (by 43%, to 4.5 mo) processes. PMA pathway devices approved on average 3 y earlier in EU, compared with 4 mo earlier for 510(k) pathway devices.	(1) EU process appears faster than FDA for high-risk devices (based on a small sample size), but only slightly faster for medium-risk devices.(2) Most FDA-approved high-risk devices are supported by advisory panels; delays in high-risk device approval are multifactorial and include multiple application amendments.
Jugo [23] (2007)	US	Descriptive analysis of 42 PMA applications submitted to FDA (2002–2007) and records of advisory committee meetings	31 of 38 applications (82%) were approved (four still pending), 29 having received positive recommendation from the advisory committee. PMA applications took an average of 1.4 y to complete, with delays due in part to numerous amendments from applicants (on average 13/application).	
Jugo [24] (2008)	US	[Same]	Among the seven rejected applications in the sample, common reasons for rejection included design flaws and statistical concerns.	
Jugo [23] (2008)	US and EU	Same, with additional comparison to EU regulatory history of same devices	Of the 42 devices, 23 confirmed to receive US and EU approval. Among these devices, EU approval was received on average 3.5 y earlier than US.	
**Studies of pre-market evaluation: classification in the US**				
GAO [25] (2009)	US	Descriptive analysis of device submissions to FDA (2003–2007)	13,199 submissions for Class I and II devicesvia the 510(k) process (90% cleared); 217 original and 784 supplemental PMA submissions for Class III devices (78% and 85% cleared, respectively). 342 submissions for Class III devices went through the 510(k) process; 228 (67%) cleared.	Many high-risk devices are still cleared through the 510(k) process; no analysis into why these mischaracterizations occurred.
**Studies of device recalls: US**				
Zuckerman et al. [26] (2011)	US	Descriptive analysis of FDA recalls (2005–2009) and approval pathways of devices that were recalled	Of 113 recalls, 80 (71%) were cleared via 510(k) process while eight (7%) had been exempted from review. 13 (16%) 510(k)-cleared devices had been designated as Class III high-risk devices, potentially more appropriate for PMA pathway.	(1) Most recalls affect devices cleared via non-PMA pathways, though majority of cleared devices are not recalled.(2) Recalls of specific devices can affect very few or millions of units/patients, making the clinical impact of recalls overall more difficult to adjudicate.(3) Some recalls may not have been avoided with more thorough pre-market review.(4) Recalls poorly measure system performance, given complexities in recognizing post-market events and translating these into FDA action
Hall [28] (2010)	US	Descriptive analysis of FDA recalls and comparison to 510(k) clearances during same time period (2005–2009)	31 of 118 recalls (28%) involved automated external defibrillators and infusion pumps. 95 (81%) were approved through the 510(k) process. During this period, 99.6% of 19,873 510(k)-approved devices did not lead to safety-related recalls.	
Maisel [29] (2010)	US	Descriptive analysis of FDA 510(k) submissions and recalls using time-to-recall as outcome measure	For approximately 3,000 devices cleared annually, recall rate of 1.3%–1.5%/year in first 4 y, 1.0%/year thereafter.	
Villarraga et al. [30] (2007)	US	Descriptive analysis of FDA recalls (2004–2006) and reasons for recall	70 (average 23.3/y) recalls involving 184 devices were adjudicated as mechanical (37%) or electrical (19%) issues. Among recalls, 57% were caused by medium-risk devices and 20% by high-risk devices.	
Battelle Memorial Institute [31] (2010)	US	Descriptive analysis of FDA recalls and comparison to 510(k) and PMA approvals during that time (2005–2010)	Recalls involved 0.16% of devices approved via 510(k) pathway and 0.85% of devices approved via PMA pathway. About half of recalls were due to pre-market design deficiencies, 29% to manufacturing problems, and 6% to labeling problems.	
GAO [32] (2011)	US	Descriptive analysis of FDA recalls (2005–2009) and interviews with FDA staff	Of 3,510 device recalls, 140 (4%) were high-risk recalls involving substantial danger for patients. Interviews revealed insufficient FDA internal review of recall episodes.	
**Studies of device recalls: EU and US/EU comparisons**				
Heneghan et al. [33] (2011)	EU	Descriptive analysis of UK recalls (2006–2010) and requests to manufacturers for data on recalled devices	2,124 Field Safety Notifications and 447 Medical Device Alerts were found, covering 197 withdrawn or recalled devices. Only 2% of manufacturers provided data for evaluating the pre-market data and safety assessments.	(1) Details of pre-market evaluation and specific safety problems are rarely accessible in EU for recalled devices.(2) Timing of high-risk recalls similar in US and EU. Rates of recalls similar on an absolute scale, but not a relative scale, given greater number of device approvals in EU
Davis et al. [34] (2011)	EU and US	Descriptive analysis of devices recalled in EU (2005–2009), results matched to US recalls, and comparisons made to existing US data	The authors estimated about 105 high-risk recalls in the EU during this period, similar on an absolute scale to the US. 45% were due to pre-market problems, comparable to rates in the US. Authors matched 126 moderate- or high-risk recalls to recalls in the US. Recall notifications posted about 3 wk earlier in the EU than in the US.	
**Surveys**				
Makower et al. [35] (2010)	US and EU	Survey of industry leaders	EU process characterized by high levels of predictability (85%, versus 22% for FDA), reasonability (91% versus 25%), and transparency (85% versus 27%). Overall, 75% of respondents viewed EU experience as excellent or very good, versus 16% for FDA experience.	(1) EU viewed as a simpler, less rigorous process, but result may be related to response bias.(2) FDA data requirements and process inefficiencies considered by industry to impede innovation and limit patient access to novel devices.(3) Surveys poorly done, limited by small sample sizes
PricewaterhouseCoopers [1] (2011)	US and EU	Structured interviews with industry leaders	FDA perceived as having more rigorous and lengthier device approval process than EU.	
PricewaterhouseCoopers [36] (2011)	US and EU	Survey of industry leaders	40% of respondents believed FDA rejected applications because of lack of resources. 63% reported FDA changed its opinion during the review process.	

### Pre-Market Evaluation (*n* = 9)

#### Empirical basis for approval (*n* = 4)

Four studies, all peer-reviewed, addressed the empirical basis for approval. Two main groups evaluated the scientific basis leading to high-risk device approval in the US. The first group, consisting of Sanket Dhruva, Lisa Bero, and Rita Redberg, reviewed 123 clinical studies contributing to applications for 78 cardiovascular devices approved via the PMA pathway from 2000 to 2007 [16]. They found that about two-thirds of the PMA applications were approved on the basis of a single study, and that the studies had a mean enrollment of 308 patients (range 23–1,548). The studies included 213 primary end points, of which 187 (88%) were surrogate measures, such as target lesion revascularization for a coronary artery stent and lead implant success for an electrophysiology device. Only 111 (52%) primary end points included comparison with controls (including 34 retrospective controls). When the authors looked at the characteristics of the 123 trials, they found that 27% were randomized, and 14% were blinded.

Two subsequent studies used the same database. Chen et al. highlighted the inclusion of “training” patients, defined in study protocols as run-in, roll-in, or investigational as part of an individual physician's or center's role in the overall pre-market clinical experience [17]. Of the main sample of 123 studies, 20 (16%) enrolled training patients, but in all cases the training patients were excluded from effectiveness analyses, and in only one study were the training patients included in safety analyses. Gender distribution was not reported in 34 (28%) of the 123 studies, and fewer than half reported gender-specific analyses or comment [18], despite FDA requirements that they do so [19].

Another group, led by one of us (D. B. K.), also reviewed clinical data submitted to the FDA during this period (2000 to 2007) for cardiovascular devices approved by the PMA pathway [20]. The authors analyzed the quality of the safety and effectiveness end points reported in the studies, defining “high quality” end points as those containing clear definitions and a specific time point for analysis. They found that effectiveness end points were high quality in 82% of trials, while safety end points were of high quality in 60%. High-quality subject accounting, defined as follow-up for ≥90% of the study cohort, was found in 77%. The studies infrequently reported key cardiovascular co-morbidities, often omitted participants' race, and rarely included pediatric patients.

The primary conclusion from these studies was that important improvements could be made in the FDA approval of Class III devices. Classic features of high-quality clinical trials—such as use of randomization, active controls, and blinding—are often absent in pre-market trials of new devices, although specific trial design elements used in drug studies may not always be appropriate or feasible for devices (e.g., blinding in a study of left ventricular assist devices).

#### Approval timing (*n* = 4)

Four studies focused on the duration of pre-market review in the US and EU. A collaboration between the California Healthcare Institute and the Boston Consulting Group, led by Gollaher and Goodall, compared the time lag between EU and US approval for a convenience sample of 12 medical device companies [21]. For these companies' 46 devices approved via PMA, the time lag averaged about 3 y, and increased from 1.2 y in 2004 (*n* = 5) to 3.9 y in 2010 (*n* = 3). For devices cleared by the 510(k) pathway, differences in EU and FDA approval times were less stark, as clearance in the US lagged by only about 4 mo (range: 1 to 9 mo). In addition, the time lag for 510(k)-cleared devices tightened from 2008 to 2010, with an average delay of only 18 d (*n* = 61; range 0 to 3 mo) for US clearance by 2010. In a subset of 105 510(k) products where the FDA did not require submission of any new clinical data, the US cleared 65 (57%) before the EU. In summary, outside of some Class III devices approved through a full PMA (which account for about 1% of all newly marketed devices), the data show limited differences in approval times. This study was not peer-reviewed, however, and its methods are obscure and subject to bias favoring the views of device industry personnel.

Ralph Jugo of Qualify First International, a medical device consultancy firm, evaluated the PMA filing and approval experience. His first article reviewed PMA submissions from 2003 to 2007 that were presented before advisory committees [22]. Jugo identified 42 total applications (38 original and four supplemental), including for circulatory system devices (26%), general and plastic surgery devices (19%), and orthopedic devices (14%). Among the 38 applications on which the FDA had made a decision, 29 (76%) received a positive recommendation from the advisory committee and were subsequently approved by the FDA, and another two (5%) received a negative recommendation from the advisory committee but were approved anyway (a total approval rate of 82%). For 31 applications for which data were available, the average review period was 513 d (16.8 mo). Twelve applications (39%) were designated for expedited review, which is generally reserved for devices that are a breakthrough technology addressing an unmet clinical need. These PMAs had an average review period of 402 d (13 mo). There were several causes for PMA approval delays, including amendments (13 filings on average per application) that provided additional information on submissions.

Jugo then evaluated the EU approval status of these same 42 devices [23]. Of the 23 that received FDA and EU approval, all received EU approval first (average time lag: 3.5 y; range 34 d to 8.5 y). An additional five devices received EU approval and were later rejected by the FDA (average time lag: 4.7 y). These calculations may be inaccurate, however, because lack of available data forced Jugo to estimate dates of receipt of EU approval for an unspecified number of devices.

Jugo's final paper assessed the seven devices that were rejected by the FDA from 2002 to 2007 [24]. Out of these seven rejections, five received EU approval. Reasons for the FDA rejection included (1) clinical study design flaws, (2) missing clinical data, (3) failure to achieve statistical significance for important end points, (4) failure to demonstrate clinical benefits, and (5) persistent safety concerns. The author did not find safety problems reported by France, Germany, or the United Kingdom (UK) related to the five devices rejected by the FDA, and concluded (based on this extremely limited sample) that the EU regulatory system has a lower burden of proof for approval and faster approval time, without risk to patient safety. A major limitation of this analysis, however, is that documents related to rejected PMA applications are not made public unless released along with other materials related to advisory panel meetings.

#### Pre-market classification in the US (*n* = 1)

A 2009 GAO report found that from 2003 to 2007, the FDA evaluated 13,199 submissions for Class I and Class II devices via the 510(k) process (90% cleared) and 217 original and 784 supplemental PMA submissions for Class III devices (78% and 85% cleared, respectively) [25]. In that same time period, the FDA reviewed 342 submissions for Class III devices through the 510(k) process, clearing 228 (67%) of these submissions. The GAO concluded that these devices needed to be either reclassified to a different device class or required to seek approval via PMA.

### Device Recalls (*n* = 8)

#### Recalls in the US (*n* = 6)

Zuckerman et al. analyzed FDA recalls from 2005 to 2009 that were designated as having the highest clinical importance (reasonable chance for serious health problems or death) [26]. Of 113 recalls, 80 (71%) had previously been cleared by the 510(k) pathway, while another eight (7%) were exempt from review. Interestingly, 13 (16%) of the 80 devices cleared by the 510(k) pathway were designated as high-risk (Class III) devices, even though Class III devices are intended to be subject to the more rigorous PMA application process [27]. The authors suggested that if some of the 510(k)-cleared devices in this sample had been subject to a PMA application process, their risks may have been identified sooner, although the small sample sizes in those studies may not have allowed these malfunctions to be detected even by the heightened PMA application review standards. In the study, one device type (automated external defibrillators) was responsible for nearly all of the recalls affecting Class III devices cleared through the 510(k) process.

In a report presented to the Institute of Medicine but not peer-reviewed, Ralph Hall identified 118 unique recalls from 2005 to 2009 that were designated by the FDA as involving the greatest safety concerns (i.e., Class I recalls) [28]. The largest subset of recalls (31, 28%) was associated with automated external defibrillators and infusion pumps. Hall found that the majority of recalled devices (95, 81%) were cleared through the 510(k) process, although he concluded that only 48% of those recalls were related to safety data that could have been identified through pre-market review. He then compared these recalls to the 19,873 510(k) applications during that time and calculated that 99.6% of 510(k) submissions did not lead to safety-related recalls. Hall concluded that additional human clinical studies would not significantly impact these recalls, given that there are few undiscovered clinical issues. However, it is highly subjective to postulate what would have been identified with pre-market review. The Hall report is also limited by its focus on only the highest risk recalls, which are less common than other types of recalls, and by his choice of a denominator that serves to diminish potential device risks. Using submissions rather than approvals as the sample further complicates his analysis. Additionally, his raw calculations of recall rates did not take into account differing marketing times for the individual devices, which may influence the likelihood of a given device being recalled.

William Maisel presented data on 510(k) submissions from 1996 to 2009 and recalls of all types (low, medium, and high risk) from 2003 to 2009 [29]. Though also not peer-reviewed, Maisel's study evaluated recalls using a more robust and statistically defensible survival analysis method than Hall [28], assessing the proportion of 510(k) clearances that were free from recall action at 1-y post-market intervals. Maisel calculated an annual recall rate of approximately 1.3%–1.5% per year for the first 4 y after clearance, and a 1.0% per year recall rate thereafter. At 5 y, 92.6% of devices were free from recall. Recalls were more common for devices with larger numbers of predicates and for life-sustaining devices cleared through 510(k).

An earlier study by Villarraga et al. analyzed a subset of recalls from 2004 to 2006 [30]. The authors identified 70 serious recalls involving 184 devices by searching through the FDA's online databases and enforcement reports. The majority of recalls were attributed to mechanical (37%) or electrical (19%) problems. Post-market issues such as manufacturing (4% of recalls), contamination (4%), or shipping (3%) represented a minority of absolute recalls. By recall number, 57% were associated with Class II (moderate risk) devices and 20% with Class III devices. This study also reported the number of units (individual products) affected by the recalls in question. Over 28 million units were subject to recall, but—as was the case in the Hall report and the Zuckerman et al. study—these were distributed unequally, as approximately 12 million units were recalled insulin pumps or blood glucometers with mechanical failures.

A non-peer-reviewed report self-published by the Battelle Memorial Institute (funded by AdvaMed, a trade association representing the medical technology industry) reviewed FDA high-priority recalls from January 2005 to May 2010 [31]. Instead of submissions, this study used total clearances as the denominator, and found that 0.16% of devices cleared along the 510(k) pathway and 0.85% of devices approved along the PMA pathway resulted in recalls. Approximately 50% of recalls were due to pre-market design deficiencies, with 29% due to manufacturing problems and 6% to labeling problems. The group concluded that the vast majority of submissions and eventual clearances and approvals emerging from the FDA do not result in serious recalls.

Finally, a 2011 GAO report evaluated the FDA's response to 3,510 device recalls occurring from 2005 to 2009 [32]. 140 recalls (4%) were characterized as high risk (those most likely to lead to patient harm). In exploring these data through interviews with FDA staff, the GAO found the lack of a systematic, timely approach to analyzing recall data, such as time-trend analyses or device- or specialty-specific analyses. The GAO also evaluated the “audit checks” associated with the high-risk recalls, which involved calls to device users from the FDA intended to confirm that they had been contacted by the manufacturer and had received instructions related to the device malfunction. Among over 2,000 audit checks, nearly 90% had inconsistencies in performance and documentation, indicating a lack of consistency in this process and making it difficult to identify whether a recall had been successfully carried out.

#### Recalls in the EU and US/EU comparisons (*n* = 2)

We found one study of device recalls in the EU. Heneghan et al. analyzed UK medical device recalls from 2006 to 2010 [33]. Using a database of public notification maintained by the Medicines and Healthcare Products Regulatory Authority, the authors found 2,124 Field Safety Notifications (disseminated by a manufacturer when a device needs to be recalled for any reason) and 447 Medical Device Alerts (disseminated by the Medicines and Healthcare Products Regulatory Authority to relate critical safety information to device users). The devices at issue ranged from high-risk devices (60, 13%) to low-risk devices (132, 30%). The 447 Medical Device Alerts covered 197 withdrawn or recalled devices, but when the authors sought information from the manufacturer—including the name of the approving body, where the device was manufactured, the clinical data supporting the device's EU approval, and details of the safety issue—about the 192 devices for which manufacturer contact information was available, only four (2%) manufacturers provided the requested data. The annual number of Medical Device Alerts stayed relatively consistent during the 5-y study period (range: 73–100), though the number of Field Safety Notifications increased by 1,220% (62 in 2006 to 757 in 2010).

Finally, we found one report (not peer-reviewed) by a management consulting firm, funded by AdvaMed, directly comparing EU and US recalls from 2005 to 2009 [34]. Since the EU lacks a centralized reporting system for adverse events related to medical devices, Davis et al. compiled a dataset of recalls based on National Competent Authority Reports, involving a process that all member companies are required to perform in reporting major safety issues for medical devices, and Field Safety Notifications, the system through which the EU member states that are most active in device safety reporting (the UK, France, Ireland, and Germany) post public notifications. After collecting EU device safety reports, two independent reviewers identified the most severe threats to public health, which would allow direct comparisons to FDA high-risk recalls. In addition, a factor of 1.66 was employed to scale up the recalls to cover the entire EU, since Field Safety Notifications from only five member states were used. In total, the authors estimated about 105 high-risk recalls in the EU for medical devices from 2005 to 2009. The study concluded that recalls for medical devices were comparable between the FDA and EU in terms of absolute number. When the authors matched 126 high- and moderate-risk recalls from the EU to similar recalls in the US, they found an even split between recalls reported first in the EU (11) and in the US (12). The EU reported more (61%) of the less severe recalls earlier than in the US. On average, notifications in the EU were posted approximately 3 wk earlier than in the US. However, the lack of an EU centralized reporting system makes it difficult to know whether all EU device recalls were captured, or to assess the accuracy of the 1.66 scale factor. Also, the thresholds for issuing recalls may not be the same in the US and EU, further complicating comparisons.

### Surveys of Device Manufacturers

We found three surveys of device manufacturers, all conducted by business consultants and not peer-reviewed. Makower et al. surveyed the opinions of 204 medical device manufacturers, representing 20% of all US device manufacturers who are “actively working on bringing innovative new medical devices to market” (but only 4% of all medical device manufacturers registered with the FDA) [35]. The survey was designed to assess respondents' experiences working with FDA and EU authorities and was funded by the Medical Device Manufacturers Association (a trade association), the National Venture Capital Association, and relevant state business associations. Respondents viewed the EU approval system in a more positive light than the FDA, agreeing that EU authorities are highly or mostly predictable (85%, versus 22% for FDA), reasonable (91% versus 25%), and transparent (85% versus 27%). Seventy-five percent of respondents viewed their overall experience with the EU as excellent or very good, compared to only 16% for the FDA. Companies reported a total cost of US$31 million per device cleared via the 510(k) pathway (with 77% [US$24 million] spent on activities related to FDA approval) compared to US$94 million per device approved via PMA application (with 80% [US$75 million] on activities related to FDA approval). The authors concluded that the FDA's clinical data requirements, extended delay times, and inefficient processes were inferior to the EU system, weakened innovation, and harmed patients.

PricewaterhouseCoopers, a global consultancy, analyzed medical technology innovation in two separate reports funded by large device trade associations. The first was based on structured interviews with executives at 13 US-based medical technology companies, whose firms represented 10% of revenue in this field. This report found that the FDA was perceived as having a more rigorous and lengthier device approval process, as compared to Germany, the UK, and France [1]. The second survey collected a convenience sample of 50 company representatives at BIOCOM, a regional conference of 550 southern California life science companies [36]. The cohort included 19 companies developing medical devices with revenues ranging from under US$10 million to over US$500 million. Forty percent of respondents believed the FDA rejected applications because of the lack of staffing resources. In addition, 63% of respondents reported that the FDA changed its opinion during the review process.

The text of the survey questions and interview guides did not accompany the Makower et al. [35] and PricewaterhouseCoopers [1],[36] reports, and independent confirmation of specific assertions was not provided, limiting interpretation of the responses. Additionally, the non-random selection of participants may have biased the responses.

## Discussion

Our systematic review of the empirical data evaluating US and EU device approval and post-market surveillance systems found quality problems in pre-market submissions in the US, provided snapshots of post-market experiences in the US and EU derived from recall analyses, and reported findings from surveys of some industry leaders.

These studies and reports provide a few reasonably firm conclusions. The FDA, which is the subject of all but one (19/20, 95%) of the studies and reports in our sample, could improve oversight of device approval in important ways. For example, the GAO report [32] and Zuckerman et al. [26] point to concerning practices that permit approval of high-risk devices based on limited evidence. In response, the FDA has committed to completing a reclassification by the end of 2012 [37]. It is also of concern that many PMA approvals in the US are based on studies with poorly defined end points, or those without blinding or randomization. Though these features are assumed to be consistent with high-quality clinical trials, they may not be realistic for studies of some devices. Better post-market surveillance of devices approved based on such limited data is necessary [8].

In general, though, the outcomes addressed by the studies in our review limit the ability to draw conclusions from them. For example, using recalls to measure the safety record of individual devices or classes of devices is flawed. Particular devices may be over- or underrepresented in recall data depending on the frequency of their use, design complexity, and the clinical manifestations of malfunction. A device malfunction must also be reported to the regulatory agency in a way that supports data aggregation and analysis. For example, the recall of the Medtronic Sprint Fidelis implantable cardioverter-defibrillator lead occurred in October 2007 due to higher-than-expected rates of lead fracture. The recall occurred 3 y after the device was approved and 3 mo after publication of a pivotal case series by Hauser et al. [38]. During the time Fidelis was on the US market, the FDA had collected 679 Fidelis-related reports, but had not yet identified a specific problem, in part because these reports were of variable quality and dwarfed by the overall number of adverse event submissions. As this case shows, the vigilance of patients, clinicians, and regulators weighs heavily in the likelihood of actually identifying device problems and potential reasons for recalls. Because the progression from device malfunction to problem recognition, analysis, reporting, and eventual recall may not be straightforward, using recall rates alone cannot judge the success or failure of a system [39].

We also found almost no data rigorously addressing device regulation in the EU, apart from a few studies evaluating the timing of approval. While case reports have suggested substantial dangers to EU patients from devices approved on the basis of limited data, no researchers have been able to systematically compare the quality of studies used for device approval or post-approval safety outcomes between the EU and US. A primary reason for this is the lack of transparency among the EU Notified Bodies, which generally do not release the data upon which they make their approval determinations. Thus, system changes that elucidate the basis for EU device approval are essential for policymakers seeking to identify the ways in which the system is functioning effectively, and to evaluate the ways it can be improved.

This systematic review has certain limitations. Despite supplementing our search with conversations with local experts in the field and searches of internal citations, we could have missed published studies or reports in this field. In addition, we found a number of studies where the data collection techniques used by the authors cast doubt on their results. For example, the surveys reviewed here are limited by biased sample selection, small response rates, recall bias and lack of independent verification of reported costs, variability in the survey responder within companies, and social desirability response bias in responses from device manufacturer representatives [40]. Few legitimate conclusions can be drawn from these surveys. As another example of the limitations of the reports, in their calculations of US and EU review times, both the Gollaher and Goodall report [21] and the Jugo studies [22]–[24] used mean times rather than medians, which may skew their results based on outlier data. In addition, the Gollaher and Goodall report used calendar days rather than actual review days, complicating comparisons. As a result, when the FDA presented time estimates in a recent performance report submitted to Congress, the agency reported that high-risk PMA-approved devices were approved after an average of 1.2 y in 2010—compared to 3.9 y in the Gollaher and Goodall report [41]. The latter figure was also derived from only three devices, further limiting its import.

Despite these limitations, this systematic review does provide some insights for policymakers seeking to reform device regulation in the US and EU, including the need for greater transparency and coordinated oversight in the EU. Yet it still remains unclear whether the US or EU approach achieves better outcomes for patients receiving devices. This assessment is further complicated by the multiple stakeholders—including patients, payors, physicians, and manufacturers—whose perspectives on system performance vary by virtue of how they weigh the importance of outcomes such as cost, speed, safety, and effectiveness. Thus, any future changes to these device approval and post-marketing systems must be accompanied by ongoing research to ensure that there is better assessment of these outcomes in both the US and EU settings. Developing such evidence can promote better use of public resources, and avoid burdensome and ineffective regulations. Until there is a more sustained commitment to developing these data, policymakers will continue to struggle to provide regulatory solutions.

## Supporting Information

Text S1 PRISMA checklist.
(DOC)

## Abbreviations

EU: European Union
FDA: US Food and Drug Administration
GAO: US Government Accountability Office
PMA: pre-market approval
UK: United Kingdom
US: United States
